# Characterization of the role of Samsn1 loss in multiple myeloma development

**DOI:** 10.1096/fba.2020-00027

**Published:** 2020-08-05

**Authors:** Natasha L. Friend, Duncan R. Hewett, Vasilios Panagopoulos, Jacqueline E. Noll, Kate Vandyke, Krzysztof M. Mrozik, Stephen Fitter, Andrew C.W. Zannettino

**Affiliations:** ^1^ Myeloma Research Laboratory Adelaide Medical School Faculty of Health and Medical Sciences University of Adelaide Adelaide Australia; ^2^ Precision Medicine Theme South Australian Health and Medical Research Institute Adelaide Australia; ^3^ Central Adelaide Local Health Network Adelaide Australia

**Keywords:** 5TGM1, bone marrow, KaLwRij, multiple myeloma, Samsn1, SAMSN1

## Abstract

The protein SAMSN1 was recently identified as a putative tumor suppressor in multiple myeloma, with re‐expression of Samsn1 in the 5TGM1/KaLwRij murine model of myeloma leading to a near complete abrogation of intramedullary tumor growth. Here, we sought to clarify the mechanism underlying this finding. Intratibial administration of 5TGM1 myeloma cells into KaLwRij mice revealed that Samsn1 had no effect on primary tumor growth, but that its expression significantly inhibited the metastasis of these primary tumors. Notably, neither in vitro nor in vivo migration was affected by Samsn1 expression. Both knocking‐out SAMSN1 in the RPMI‐8226 and JJN3 human myeloma cell lines, and retrovirally expressing SAMSN1 in the LP‐1 and OPM2 human myeloma cell lines had no effect on either cell proliferation or migration in vitro. Altering SAMSN1 expression in these human myeloma cells did not affect the capacity of the cells to establish either primary or metastatic intramedullary tumors when administered intratibially into immune deficient NSG mice. Unexpectedly, the tumor suppressive and anti‐metastatic activity of Samsn1 in 5TGM1 cells were not evidenced following cell administration either intratibially or intravenously to NSG mice. Crucially, the growth of Samsn1‐expressing 5TGM1 cells was limited in C57BL/6/Samsn1^−/−^ mice but not in C57BL/6 Samsn1^+/+^ mice. We conclude that the reported potent in vivo tumor suppressor activity of Samsn1 can be attributed, in large part, to graft‐rejection from Samsn1^−/−^ recipient mice. This has broad implications for the design and interpretation of experiments that utilize cancer cells and knockout mice that are mismatched for expression of specific proteins.

## 1 INTRODUCTION

Cancer is the second leading cause of death worldwide and accounted for an estimated 9.6 million deaths in 2018 (www.who.int/health‐topics/cancer). Multiple myeloma (MM) is a cancer characterized by the clonal proliferation of antibody‐secreting plasma cells (PCs) within the bone marrow (BM).^1^, ^2^ MM typically presents with numerous sites of malignant PC tumors at numerous sites throughout the axial and appendicular skeleton and clinical manifestations include hypercalcaemia, renal insufficiency, anemia and lytic bone lesions, also referred to as the so called CRAB symptoms.^3^ Patients with MM have a median age of diagnosis of 66‐70 years,^4^ and the disease constitutes 10% of all hematological malignancies and 1% of all cancers,^5^ with over 30,000 new cases annually diagnosed in the United States.^6^ Most cases of MM have evolved from the largely asymptomatic premalignant condition known as monoclonal gammopathy of undetermined significance (MGUS), characterized by a lower PC burden and absence of end‐organ damage.^7^ MGUS is common in the aging population, with an estimated frequency of 2%–3% in persons over 49 years of age.^8^ Individuals with MGUS have a continual risk of 1% per annum of their disease evolving to diagnostic MM.^9^ Despite recent therapeutic advances MM patients can still only expect 5‐year survival rates of close to 50%.^10^

Like most cancers MM is genetic in origin, but no single genetic mutation causes the disease. MM is genetically heterogeneous, with the tumor cells from each patient having a unique combination of single nucleotide variants (SNVs), copy number abnormalities (CNAs), aneuploidies and translocations.^11^ Some of the more frequent mutations include: trisomies of chromosomes 3, 5, 7, 9, 11, 15, 19 and 21 ^12^; translocations involving the immunoglobulin heavy chain (IgH) locus at chromosome 14q32^13^; gain of chromosome 1q^14^; loss of chromosome 13q^15^; and mutation of the *NRAS*, *KRAS* and *BRAF* genes.^16^ MM PCs also have significantly different transcriptional profiles to normal PCs, often associated with global epigenetic changes,^17^ dysregulation of transcription factors,^18^ and localized genomic copy number variants.^19^ Detailed characterization of genomic deletions, epigenetically silenced regions and gene expression levels have highlighted many putative tumor suppressor genes in MM.^20^, ^21^, ^22^

One such tumor suppressor gene is *SAMSN1*, which was found to have reduced expression in several different cancer types. The first description of reduced *SAMSN1* expression was in lung cancer, in which loss of heterozygosity at 21q21, the chromosomal location of the *SAMSN1* gene, is a common abnormality.^23^ In addition, ulcerative colitis patients with colon cancer were found to have significantly lower expression of *SAMSN1* compared to those patients without cancer, suggesting that SAMSN1 may inhibit the transition from pre‐neoplastic lesions to overt malignancy in colorectal cancer.^24^ Furthermore, *SAMSN1* mRNA expression was found to be lower in cancerous tissues compared to normal adjacent tissue from gastric cancer and hepatocellular carcinoma patients.^25^, ^26^ Low *SAMSN1* expression in these cancers was found to be associated with increased tumor size and decreased overall survival, suggesting that *SAMSN1* may also be a tumor suppressor gene in gastric cancer and hepatocellular carcinoma.^25^, ^26^

SAM domain, SH3 domain and nuclear localization signals 1 (*SAMSN1*), also known as *SASH2*/*NASH1*/*HACS1*/*SLy2*, was first identified in a study of genes expressed in MM and is localized on human chromosome 21 (q21.1).^27^ *SAMSN1* is highly expressed in the hematopoietic compartment, including peripheral blood lymphocytes, immune tissues and the BM, and to a lesser extent in other tissues, including the heart, lung and brain.^27^, ^28^ It is a putative cytoplasmic adaptor protein that is significantly upregulated following B cell activation,^29^, ^30^ and overexpression of Samsn1 in murine splenic cells inhibits proliferation in response to activating stimuli.^29^ Conversely, increased B cell and T‐cell proliferation in vitro and enhanced humoral immune responses in vivo were observed in *Samsn1* ^−/−^ mice compared to WT mice.^31^ As well as these roles in limiting lymphocyte proliferation, SAMSN1 has also been implicated in the control of actin cytoskeleton remodeling, a process involved in cell adhesion and migration.^32^, ^33^ Identified binding partners of SAMSN1 include proteins involved in the regulation of B cell activation (the paired immunoglobulin‐like receptor B, PIR‐B),^34^ actin polymerization (cortactin, Hs1),^32^, ^35^ and transcriptional repression (Sin3 co‐repressor complex proteins).^36^

Specifically in relation to MM, we and others have previously shown that expression of the SAMSN1 gene is significantly decreased in MM PCs compared to MGUS or normal PCs.^37^, ^38^ Moreover, C57BL6/KaLwRij strain mice, which have a predisposition to develop an MM‐like malignancy in old age,^39^, ^40^ were shown to harbor a large 180 kb genomic deletion that completely removes the *Samsn1* coding sequence.^37^, ^38^ Restoration of Samsn1 expression in the C57BL6/KaLwRij‐derived myeloma cell line 5TGM1 led to a remarkable abrogation of the capacity of these cells to produce bone marrow (intramedullary) tumors in vivo.^37^ These data were consistent with SAMSN1 having a substantial tumor suppressor role in human MM. Here, using a panel of SAMSN1/Samsn1 knockdown and transgenic cell lines and multiple mouse strains, we set out to further investigate the conditions under which SAMSN1 expression so potently abolishes tumor growth in vivo.

## 2 MATERIALS AND METHODS

### 2.1 Cell culture

Unless otherwise specified, all cell culture reagents were sourced from Sigma‐Aldrich and all media were supplemented with 2 mmol/L L‐glutamine, 100 U/ml penicillin, 100 µg/ml streptomycin, 1 mmol/L sodium pyruvate, and 10 mmol/L HEPES buffer. All cell lines were tested for mycoplasma infection using a MycoAlert^TM^ Mycoplasma Detection Kit (Lonza). Human myeloma cell line (HMCL) RPMI‐8226 was purchased from the American Type Culture Collection (ATCC), while the HMCLs LP‐1, OPM2 and JJN3 were a kind gift from Prof. Andrew Spencer (Monash University, Australia). All HMCLs were maintained in Roswell Park Memorial Institute 1640 (RPMI‐1640) medium with 10% fetal calf serum (FCS, Thermo Fisher Scientific). The murine MM 5TGM1 PC line was originally kindly provided by Assoc Prof Claire Edwards (University of Oxford, UK). 5TGM1 cells expressing both green fluorescent protein (GFP) and luciferase were previously generated using the retroviral expression vector NES‐TGL.^41^ 5TGM1 cells were maintained in Iscove's Modified Dulbecco's Medium (IMDM) with 20% FCS. Bone marrow stromal cells (BMSCs) were isolated by plastic adherence from bone chips of healthy adult KaLwRij mice. Thawed BMSCs were seeded in Minimum Essential Medium Eagle, Alpha Modification (α‐MEM) with 10% FCS and 100 mmol/L L‐ascorbate‐2‐phosphate. A transformed human BM endothelial cell (TrHBMEC^42^) line was kindly provided by Prof. Babette Weksler (Cornell University, USA). TrHBMECs were maintained in gelatin‐coated flasks and Medium 199 with 20% FCS and supplements consisting of 0.1% sodium bicarbonate, 1 mmol/L sodium pyruvate, 20 mmol/L HEPES, 50 U/ml penicillin, 50 μg/ml streptomycin, 1× non‐essential amino acids, 50 µg/ml heparin and 100 µg/ml endothelial cell growth supplement (BD Biosciences).

### 2.2 Mouse colonies

C57BL/6 J (“C57BL/6”) and NOD.Cg‐Prkdc*^scid^*Il2rg*^tmlWjl^*/SzJ (“NSG”) mice were originally obtained from The Jackson Laboratory (Maine). C57BL/KaLwRij.Hsd (“KaLwRij”) mice (Envigo) were obtained from Prof. Andrew Spencer (Monash University, Australia). All mice were held in SAHMRI Bioresources Facility under Specific Pathogen Free conditions. C57BL/6/Samsn1^−/−^ mice were generated by 10 rounds of backcrossing KaLwRij mice on to the C57BL/6 J strain. Genotyping was performed using PCR primers spanning the *Samsn1*‐deletion, as previously described.^37^ All animal experiments were conducted under SAHMRI Animal Ethics Committee Project SAM165.

### 2.3 Mouse models of myeloma

For intratibial (i.t.) models, NSG or KaLwRij mice between 5 and 6 weeks of age received an i.t. injection of 10 µL of cell suspension (5 × 10^5^ cells per inoculum in phosphate buffered saline (PBS) for HMCLs, 1 × 10^5^ cells per inoculum in PBS for 5TGM1 cells) as per Cheong, et al.^43^ The endpoint of the experiment was determined based on the first sign of morbidity (3 weeks for OPM2 and JJN3, 5 weeks for RPMI‐8226, 8 weeks for LP‐1, and 23 days for 5TGM1). For intravenous (i.v.) models, 5 × 10^5^ 5TGM1 cells in 100 µL PBS were injected via the tail vein into 6‐8 week old KaLwRij, NSG, C57BL/6, or C57BL/6‐Samsn1^−/−^ mice. For short‐term in vivo migration assays, 5 × 10^6^ cells in 100 µL PBS were injected via the tail vein as per Opperman, et al.^44^ Tumor burden for HMCLs was measured at experimental endpoints by flow cytometry. Serum protein electrophoresis, whole animal bioluminescence, flow cytometry, and immunohistochemistry were used to quantitate 5TGM1 tumor burden at regular intervals, as indicated.

### 2.4 Tumor monitoring by bioluminescence imaging (BLI) or flow cytometry.

To measure tumor burden, the mice were administered firefly D‐Luciferin substrate (30 mg/ml in PBS, Biosynth) by intraperitoneal injection at a concentration of 150 mg/kg. After 10 min, the bioluminescence was quantitated using the IVIS^®^ Spectrum In Vivo Imaging System and Living Image^®^ Software v4.5.5 (Perkin Elmer).^45^, ^46^ Both whole body BLI and discrete regional BLI from selected hindlimbs was used, as indicated. For enumeration of GFP^+^ cells, BM was collected from femora and tibiae by flushing the bones with 5 ml of chilled PFE (PBS, 2% FCS, 2 mM EDTA) buffer using a 21 G needle. Long bones were cut and scraped and the combined endosteal and BM cells were filtered (70 µm filter), pelleted, resuspended in PFE and immediately analyzed for GFP^+^ tumor cells by flow cytometry on the FACSCanto^TM^ II (BD Biosciences) using FACSDiva^TM^ software v8.0 (BD Biosciences). BM from a non‐tumor mouse was used as a negative control for gating purposes.

### 2.5 Serum protein electrophoresis (SPEP)

The serum was collected following centrifugation of clotted peripheral blood at 2,000× *g* and 4°C for 10 min. M protein/paraprotein were assessed by serum protein electrophoresis (SPEP) using the Hydragel Protein (β1‐β2) 30 Kit (Sebia), according to the manufacturer's instructions. The stained SPEP gels were imaged on a Gel Doc^TM^ XR+Imager (Bio‐Rad), and the intensity of the paraprotein band/M‐spike was quantitated and normalized to the albumin band using Image Lab Software v6.0.1 (Bio‐Rad).

### 2.6 Immunohistochemistry

Tibiae that were directly injected with 5TGM1 cells were collected from KaLwRij mice at the experimental endpoint (day 23) and fixed in 10% buffered formalin. Decalcified bones were paraffin embedded and 5 µm deparaffinized sections were stained with hematoxylin and eosin (H&E) or an anti‐GFP antibody. For anti‐GFP staining, endogenous peroxidase activity was neutralized by incubation with 0.5% H_2_O_2_ in methanol for 30 min before sections were incubated with immunohistochemistry (IHC) blocking buffer (3% normal horse serum in PBS) at room temperature for 2 h. The slides were incubated with a goat anti‐GFP monoclonal antibody (#A600‐101‐215, Rockland) at 1:5,000 in IHC blocking buffer at 4°C overnight. After washing in 1x PBS, the slides were incubated with a biotinylated rabbit anti‐goat IgG antibody (#BA5000, Vector Lab) 1:250 in IHC blocking buffer at room temperature for 30 min. This was followed by incubation with a streptavidin‐peroxidase conjugate (Thermo Fisher Scientific) at 1:100 in blocking buffer at room temperature for 1 hour. The bound antibody was then visualized by incubating the slides with 3,3'‐diaminobenzidine (Sigma‐Aldrich) at room temperature in the dark for 10 min. Slides were briefly counterstained with hematoxylin solution and mounted with DePex. Slides were imaged on a BX53 microscope (Olympus).

### 2.7 WST‐1 proliferation assay

HMCLs were plated at 1 × 10^5^ cells/ml in triplicate in 100 μl of complete RPMI‐1640 medium in 4 replicate 96‐well plates and incubated at 37°C with 5% CO_2_. Every 24 h from day 0 to 3, 10 μl of WST‐1 Reagent (Roche) was added to all the relevant wells of one plate, which was then returned to the incubator for 2 h. Following incubation, the absorbance of each well at 450 nm was measured using the iMark^TM^ Microplate Absorbance Reader (Bio‐Rad). The background was subtracted from the absorbance values and the fold‐change in absorbance was calculated relative to day 0.

### 2.8 *SAMSN1* gRNA expression vectors

The MIT CRISPR design tool (http://crispr.mit.edu) was used to select two guide RNAs (gRNAs) targeting exon 4 of *SAMSN1*. The sequences of gRNA #1 and #2 were *5′*‐GGTCACTGTTTCTATATGGG‐*3′* and *5′*‐GAGACTATCCATGGAGTCAC‐*3′*, respectively. To clone the individual gRNAs, 24 bp complementary oligonucleotides containing the gRNA sequence and a 4‐bp overhang (forward: TCCC and reverse: AAAC) were annealed, phosphorylated and cloned into the *Bsmb*I‐digested FgH1tUTG lentiviral vector ^47^ (a gift from Marco Herold (Addgene plasmid # 70183)).

### 2.9 Generation of SAMSN1‐knockdown (KD) human myeloma cell lines

RPMI‐8226 and JJN3 HMCLs constitutively expressing Cas9 were generated by transducing cell lines with the FUCas9Cherry lentiviral vector^47^ (Addgene plasmid #70182), which was a kind gift from Marco Herold (WEHI, Australia). Lentiviral particles were produced in HEK293 T cells following Lipofectamine‐2000 ^TM^ (Invitrogen) transfection with the psPAX2 lentiviral packaging plasmid (Addgene plasmid #12260, a gift from Didier Trono) and the pCMV‐VSV‐G envelope protein‐expressing plasmid (Addgene Plasmid #8454)^48^ and Cherry^+^ cell lines were established. The Cas9‐expressing HMCLs were then similarly transduced with an inducible gRNA‐containing or empty pFH1tUTG vector. GFP^+^mCherry^+^ cells were isolated by FACS and gRNA expression was transiently induced by treating the HMCLs with doxycycline (Sigma‐Aldrich) at a final concentration of 1 µg/ml for 72 h.

### 2.10 Heteroduplex mobility assay

DNA was extracted from CRISPR‐targeted cells using a DNeasy^®^ kit (QIAGEN). PCR was used to amplify a 1.1 kb region encompassing exon 4 of *SAMSN1* using primers F: 5′‐CTAGGTGGCAAGCATGGTATTAGATTTG‐3′ and R: 5′‐AGAAAGAAAGAGACAGAGAATGGAGCAG‐3′. PCR products were incubated at 95°C for 5 min and the temperature was then reduced to 85°C at a ramp rate of 51%, followed by a decrease to 25°C at a ramp rate of 2.6%. The products were resolved by gel electrophoresis within a 12% polyacrylamide gel in 1x TBE buffer (100 mmol/L Tris base, 100 mmol/L boric acid, 2 mmol/L EDTA) and post‐stained with GelRed^®^ (Biotium).

### 2.11 Generation of Samsn1/SAMSN1 transgenic cell lines

5TGM1‐Samsn1 and 5TGM1‐EV (empty vector) cells were previously generated by retroviral transduction with pRufimCh2 retroviruses as detailed in Noll, et al,^37^ OPM2 and LP‐1 cells were also transduced with previously constructed pRufiG2‐EV and pRufiG2‐SAMSN1 retroviruses.^37^ Briefly, HEK293 T cells were transfected with 5 μg of either the SAMSN1 encoding‐ or empty‐ pRUFiG2 plasmid, and 5 μg of the amphotropic packaging plasmid pEQPAM3^49^ using Lipofectamine‐2000 ^TM^ (Invitrogen) according to manufacturer's instructions. GFP^+^ transduced HMCLs were isolated by flow cytometry.

### 2.12 Western blotting

Whole cell protein lysates were prepared from PBS‐washed cells using radioimmunoprecipitation assay (RIPA) buffer (1% NP‐40, 20 mmol/L HEPES, 150 mmol/L NaCl, 10% glycerol, 2 mmol/L Na_3_VO_4_, 10 mmol/L Na_4_P_2_O_7_, 2 mmol/L NaF, and 1x cOmplete^TM^ EDTA‐free Protease Inhibitor Cocktail (Roche)). Clarified lysates were quantitated using the *RC DC* ^TM^ Protein Assay Kit (Bio‐Rad), according to manufacturer's instructions. Proteins were resolved on 10% SDS‐polyacrylamide gel electrophoresis (PAGE) gels in Tris‐Glycine‐SDS running buffer (0.3% Tris‐HCl, 1.44% glycine, 0.1% SDS). Proteins were membrane‐transferred in 192 mmol/L Tris, 25 mmol/L glycine, 20% methanol, 0.02% SDS at 100 V and 4°C for 1 h. Blocked membranes were probed overnight (4°C) with primary antibodies: (rabbit polyclonal anti‐SAMSN1 antibody (cat. # HPA010645; Sigma‐Aldrich) 1:500 dilution; rabbit polyclonal anti‐HSP90 antibody (cat. #7947; Santa Cruz Biotechnology) 1:2,500 dilution; mouse monoclonal anti‐β actin antibody (cat. #A1978; Sigma‐Aldrich) 1:2,500. Blots were washed in Tris‐buffered saline with 0.1.% Tween‐20 (TBS‐T) blots and incubated with an appropriate DyLight‐680/800‐conjugated secondary antibody (Thermo Fisher Scientific) diluted 1:10,000 in TBS‐T, for 1 h. TBS‐T washed blots were then imaged using the Odyssey^®^ CLx Imager (LI‐COR). Quantitative analysis of band intensity was performed using ImageJ software (http://fiji.sc).

### 2.13 Quantitative PCR

Total RNA was extracted from HMCLs using TRIzol^TM^ reagent (Invitrogen) and isopropanol precipitation according to manufacturer's recommendations. cDNA was primed with both random hexamers and oligodT and synthesized from 2 µg total RNA using Superscript^TM^ IV reverse transcriptase (Invitrogen) in a 20 µl volume. The cDNA reaction was incubated at 23°C, 55°C, and 80°C for 10 min each. qPCR was performed, with each 15 μl reaction containing 2 μl of cDNA, 1x RT^2^ SYBR^®^ Green qPCR Mastermix (QIAGEN), 0.5 μmol/L forward primer, 0.5 μmol/L reverse primer. The following primers were used: *ACTB*‐F 5′ TTGCTGACAGGATGCAGAAG, 3′ *ACTB*‐R 5′ AAGGGTGTAAAACGCAGCTC 3′, *SAMSN1*‐ F 5′ TCCCTCAAAGCCAGTGACTC 3′, *SAMSN1*‐R 5′ GCCACAGAATGGTCCTGAAT 3′. Reactions were performed on the CFX Connect^TM^ Real‐Time PCR Detection System (Bio‐Rad) using the following cycling parameters: 50°C for 2 min; 95°C for 15 min; 40 cycles of 95°C for 15 seconds, 60°C for 25 seconds and 72°C for 10 seconds; and 72°C for 3 min. Standard curves were generated to determine the reaction efficiency of each primer pair. Normalization and relative expression analysis were performed, with the reaction efficiency taken into account, using Q‐Gene software.^50^

### 2.14 Cell adhesion assay

TrHBMECs (1 × 10^4^ cells/well) were plated in black‐walled and clear‐bottomed 96‐well plates and allowed to adhere overnight. 5TGM1 cells (1 × 10^5^ cells/well) in complete IMDM were then overlaid onto the TrHBMECs and allowed to adhere for 15 min. Following 3 media washes, the number of adherent 5TGM1 cells in each well was enumerated by the addition of 0.3 mg/ml D‐luciferin (Biosynth) followed by bioluminescence imaging using the IVIS^®^ Spectrum (PerkinElmer). The bioluminescent signal from adhered 5TGM1 cells was normalized to the signal from the total cell input.

### 2.15 Migration assays

For transwell assays, 5 × 10^5^ 5TGM1 cells in serum‐free IMDM were seeded in 8 µm transwells (COSTAR) in triplicate. The cells were allowed to migrate toward the lower chamber containing serum‐free IMDM plus 5% primary KaLwRij BMSC‐conditioned medium for 24 h. For transendothelial assays, 1 × 10^4^ TrHBMECs were plated on gelatin‐coated transwells and allowed to adhere for 24 h. HMCLs (5 × 10^5^ cells) in RPMI‐1640 medium with 1% FCS were then seeded into the BMEC‐coated transwells in triplicate. The cells were allowed to migrate toward the lower chamber containing RPMI‐1640 medium with either 20% FCS or 1% FCS and 100 ng/ml CXCL12 for 20 h. Following the incubation period, the transwells were discarded and the numbers of migrated cells present in the plate were enumerated using an Olympus CKX41 inverted light microscope and ImageJ software (http://imagej.nih.gov/ij/).

### 2.16 Data analysis

Unless otherwise described, statistical analysis was performed using GraphPad Prism v8.0.0 (GraphPad Software). The Fisher's exact test was used to determine whether the proportions of one categorical variable were different depending on the value of the other categorical variable. When three or more groups were being compared for a single variable, a parametric one‐way ANOVA with Tukey's post‐hoc multiple comparisons test or a non‐parametric Kruskal‐Wallis test with Dunn's multiple comparisons test was used. For time‐course experiments, groups were compared using a two‐way ANOVA with Sidak's or Tukey's multiple comparisons test. When two groups were being compared for a single variable, a parametric paired t test, a parametric unpaired t test or a non‐parametric Mann–Whitney U test was used. Differences were statistically significant when *p* < 0.05.

## 3 RESULTS

### 3.1 Samsn1 inhibits metastasis but not primary tumor growth in vivo

**FIGURE 1:**
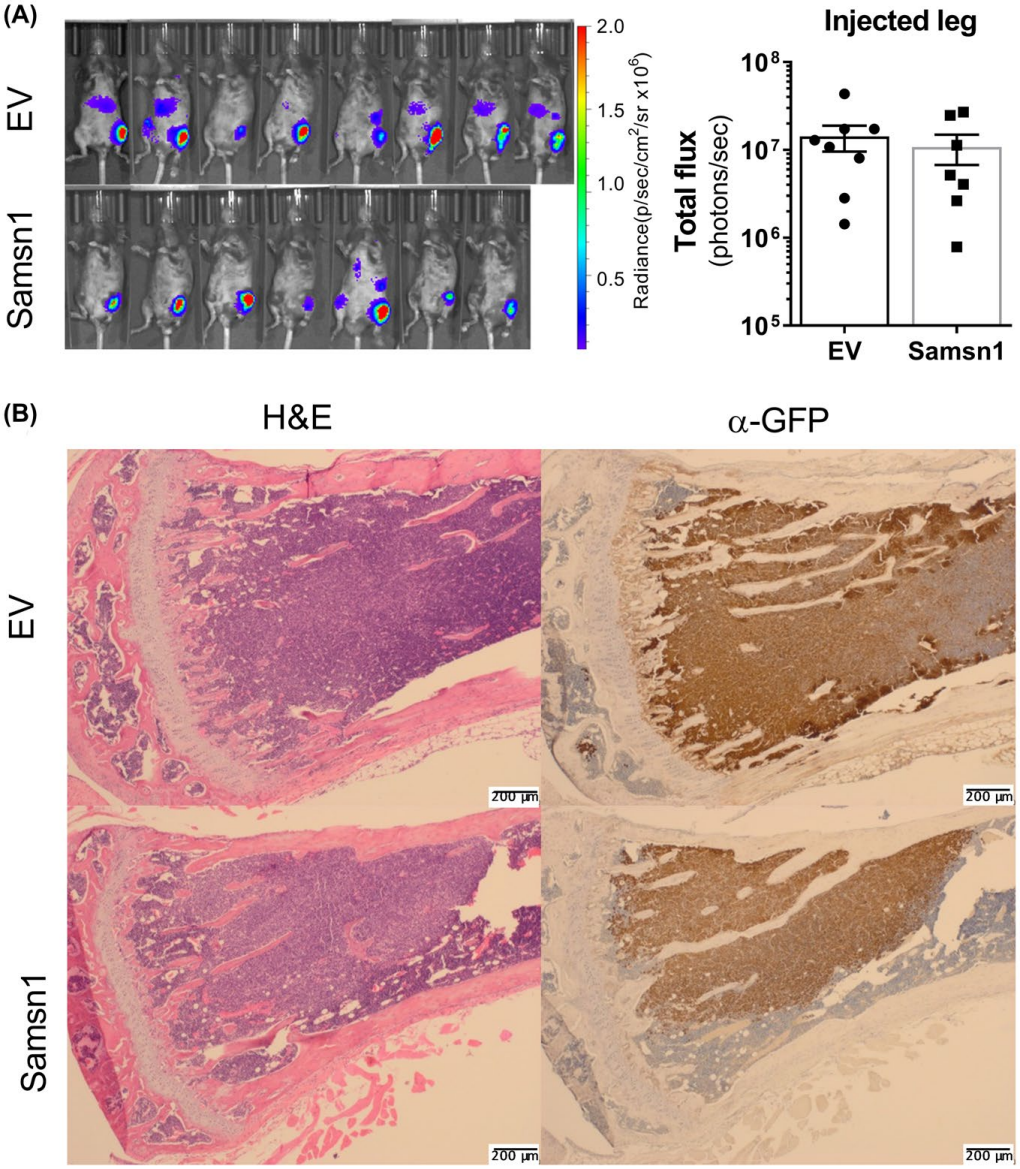
Samsn1 does not affect the growth of primary tumors following i.t. injection of 5TGM1 cells in vivo. 5TGM1‐Samsn1 (Samsn1) or 5TGM1‐EV (EV) cells were injected into the left tibia of KaLwRij mice and tumor burden was measured by BLI. (A) Ventral BLI scans of mice injected with 5TGM1‐EV or 5TGM1‐Samsn1 cells, and the quantitated total flux of the injected legs, after 23 days are shown. Graph depicts the mean ± SEM of *n* = 7‐8 mice per cell line from two independent experiments. *p* > 0.05, Mann–Whitney *U* test. (B) Paraffin‐embedded sections of the 5TGM1‐injected tibiae were stained with either H&E or an anti‐GFP antibody from mice injected with either 5TGM1‐EV or 5TGM1‐Samsn1cells

**FIGURE 2:**
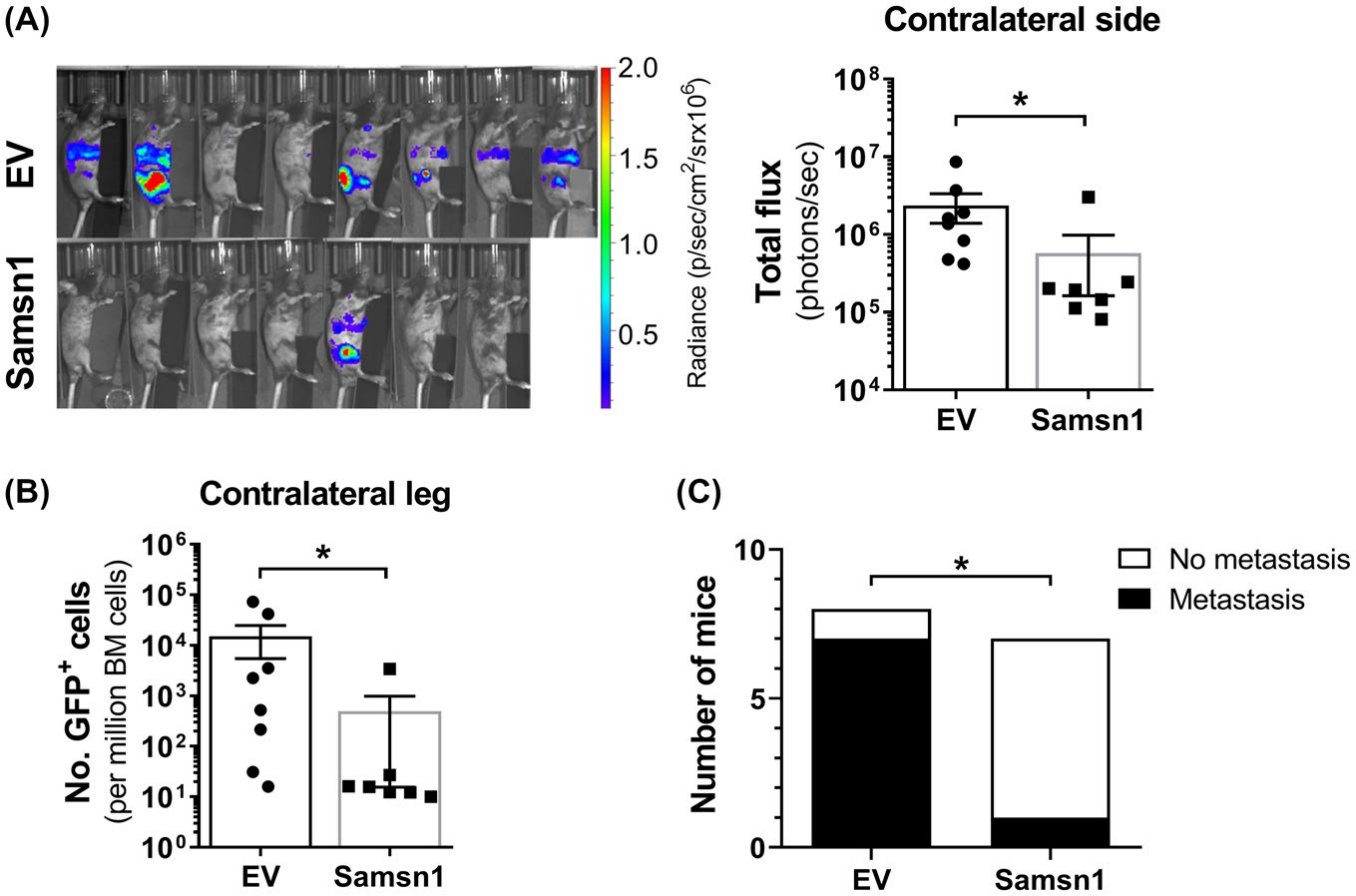
Samsn1 inhibits the metastasis of 5TGM1 cells in vivo. 5TGM1‐Samsn1 or 5TGM1‐EV cells were injected into the left tibia of KaLwRij mice and tumor burden was measured by BLI and flow cytometry. (A) BLI scans of the contralateral side (injected leg covered) of the mice inoculated with 5TGM1‐EV or 5TGM1‐Samsn1 cells and the quantitated total fluxes after 23 days are shown. (B) The number of GFP^+^ tumor cells in the BM from the non‐injected, contralateral leg was assessed by flow cytometry after 23 days. (C) The number of mice injected i.t. with 5TGM1‐EV or 5TGM1‐Samsn1 cells with overt metastasis, defined as visible BLI signal from sites other than the injected leg and/or greater than 200 tumor cells per million in the BM of the contralateral leg by flow cytometry. Results were normalized to primary tumor burden and graphs depict the mean ± SEM of *n* = 7‐8 mice per cell line from two independent experiments. *p < 0.05, Mann–Whitney *U* test (A and B) or Fisher's exact test (C)

The 5TGM1/KaLwRij mouse model is a commonly used immune competent, syngeneic, preclinical model of multiple myeloma. The 5TGM1 myeloma cancer cell line is derived from a spontaneous tumor in a C57BL/KaLwRijHsd (“KaLwRij”) mouse. We have genetically modified the 5TGM1 to express GFP and firefly luciferase which enables enumeration of tumor burden by flow cytometry and whole animal bioluminescence imaging (BLI) respectively. In addition, the 5TGM1 cells secrete a monoclonal antibody that can readily be detected in the serum of mice with established tumors as an “M‐spike” following electrophoresis of serum proteins. Upon reinjection into the tail veins of KaLwRij mice the cells migrate to the bone marrow and form multiple tumors and recapitulate many of the features of clinical MM. Alternatively, the 5TGM1 cells can be injected directly into the bone marrow space of the tibia, and the cells grow in the injected tibia as well as readily disseminating and forming metastatic myeloma tumors in the bone marrow of the non‐injected leg. Expression of Samsn1 in 5TGM1 myeloma cancer cells has previously been shown to greatly reduce the establishment of BM tumors in KaLwRij mice when the cells were administered via the tail vein.^37^ This phenomenon was consistent with Samsn1 either reducing the efficiency of cancer cell migration to the BM or reducing the growth of migrated cancer cells in the BM microenvironment. To determine the effect of Samsn1 on the subsequent growth of 5TGM1 cells in the BM without the prerequisite of tumor cells homing from the circulation, 5TGM1‐Samsn1 or 5TGM1‐EV cells were injected directly into the left tibiae of KaLwRij mice. After 23 days, the primary tumor burden in the injected leg was not found to significantly differ between the mice inoculated with 5TGM1‐Samsn1 cells and mice inoculated with 5TGM1‐EV cells, as determined by BLI (*p* = 0.5907, Mann–Whitney *U* test; Figure 1A). In addition, the formation of bone marrow (intramedullary) tumors by both 5TGM1‐Samsn1 and 5TGM1‐EV cells was confirmed by performing immunohistochemical staining of GFP^+^ tumor cells in sections from injected tibiae (Figure 1B). Notably, in some intratibially inoculated mice, the BLI showed that 5TGM1 cells had migrated from the injected leg and formed secondary tumors at distal bone sites (Figure 2A). The metastatic tumor burden was significantly lower in the 5TGM1‐Samsn1 group of mice compared to 5TGM1‐EV group of mice, as measured by BLI (*p* = 0.0093, Mann–Whitney U test; Figure 2A). In addition, the number of GFP+5TGM1 tumor cells in the BM of the femora and tibiae from the non‐injected, contralateral legs was significantly lower in the 5TGM1‐Samsn1‐inoculated mice compared to the 5TGM1‐EV‐inoculated mice (*p* = 0.0140, Mann–Whitney *U* test; Figure 2B). Collectively, both the BLI and flow cytometry data suggest that the incidence of metastasis was significantly lower in mice inoculated with 5TGM1‐Samsn1 cells (*n* = 1/7, 14.3%) compared to mice inoculated with 5TGM1‐EV cells (*n* = 7/8, 87.5%) (*p* = 0.0101, Fisher's exact test; Figure 2C). Taken together, these data suggest that Samsn1 does not affect the growth of primary tumors following i.t. injection of 5TGM1 cells into KaLwRij mice, but it significantly inhibits the subsequent metastasis of MM PC from these primary tumors.

### 3.2 Samsn1 expression in 5TGM1 cells does not affect homing to, but inhibits expansion within, the bone marrow in vivo

**FIGURE 3:**
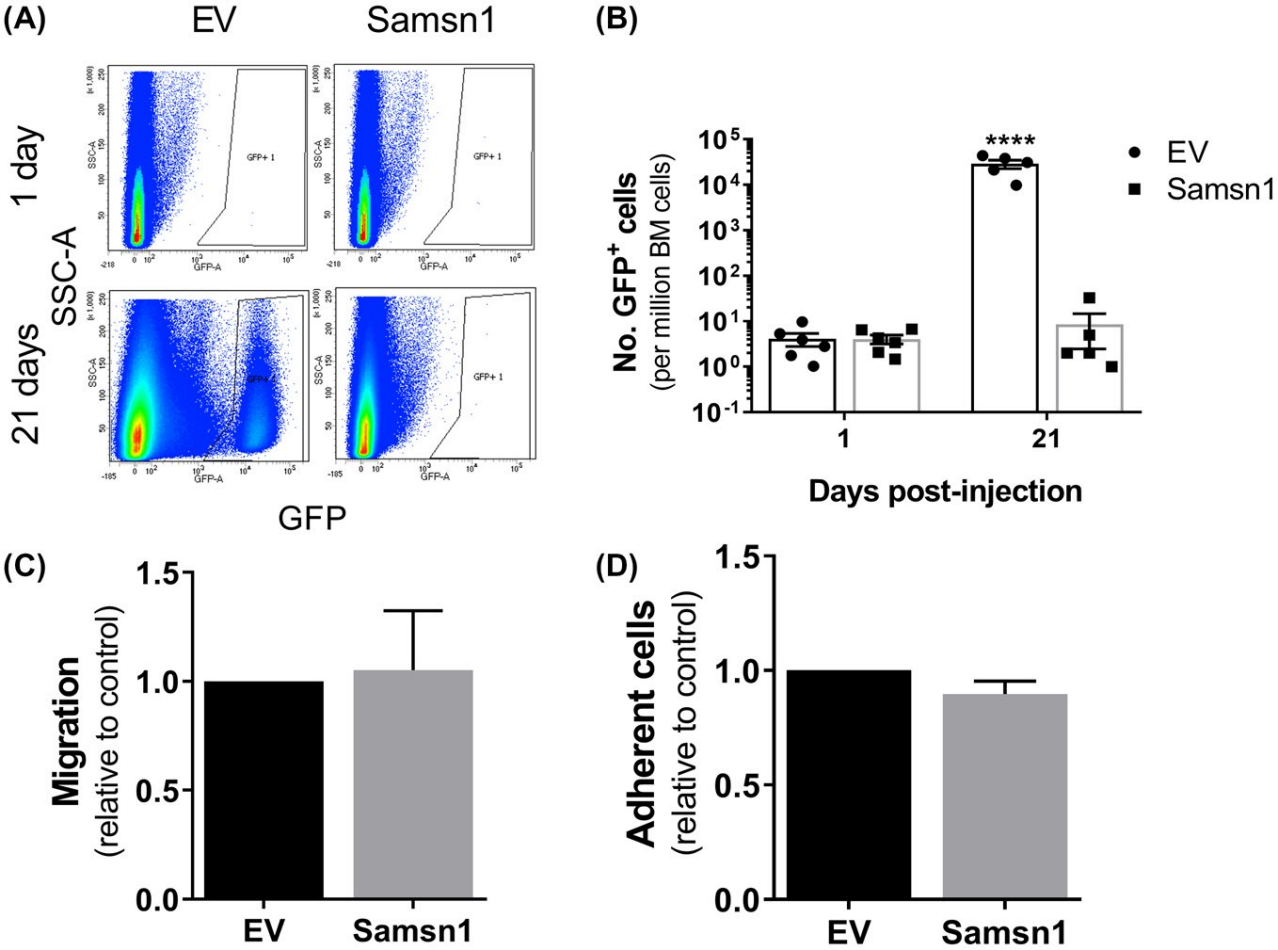
Samsn1 does not affect either 5TGM1 migration or BM homing, but does inhibit the expansion of BM‐migrated 5TGM1 cells. KaLwRij mice were injected with 5TGM1‐Samsn1 or 5TGM1‐EV cells i.v. and the number of GFP^+^ tumor cells in the long bones was determined by flow cytometry after 1 or 21 days. (A) Representative flow plots of GFP^+^ cells in the BM of mice inoculated with 5TGM1‐EV or 5TGM1‐Samsn1 cells after 1 day or 21 days are shown. (B) Graph shows the number of GFP^+^ tumor cells per million BM cells present in the long bones of mice injected with 5TGM1‐EV or 5TGM1‐Samsn1 cells after 1 and 21 days. Graph depicts the mean ± SEM of *n* = 5‐6 mice per cell line at each time point from one (21 days) or two (1 day) independent experiments. ****p < 0.0001, two‐way ANOVA with Sidak's multiple comparisons test. (C) Migration of 5TGM1‐Samsn1 and 5TGM1‐EV cells toward primary mouse BM stromal cell‐conditioned medium was assessed in a 24‐hour transwell assay. Results are expressed relative to the EV control cells. (D) 5TGM1‐Samsn1 or 5TGM1‐EV cells were seeded on a BM endothelial cell monolayer, and percent cell adhesion, relative to total cell input, was assessed by BLI after 15 min. Results are expressed relative to the EV control cells. Graphs depict the mean + SEM of six (C and D) independent experiments. *p* > 0.05, paired t test (C and D)

Given that Samsn1 was found to inhibit the metastasis of 5TGM1 cells from primary tumors, it was hypothesised that Samsn1 suppresses the homing of MM PCs to the BM. To test this in vivo, 5TGM1‐Samsn1 cells or 5TGM1‐EV cells were injected i.v. into KaLwRij mice and the number of GFP^+^ tumor cells present in the BM after 24 h was assessed by flow cytometry. Notably, Samsn1 expression was not found to affect the number of 5TGM1 cells present in the femora and tibiae of the mice 24 h post‐tumor cell injection (*p* = 0.8182, Mann–Whitney U test; Figure 3A,B). To determine the fate of the 5TGM1‐Samsn1 cells that successfully homed to the BM, the experiment was repeated, but the number of tumor cells in the hind legs of the mice was assessed after 21 days. While the numbers of 5TGM1‐EV cells in the BM expanded over time, the numbers of 5TGM1‐Samsn1 cells did not significantly differ between day 1 and 21 post‐tumor cell injection (*p* < 0.0001, two‐way ANOVA with Sidak's multiple comparison test; Figure 3A,B). The impact of Samsn1 expression on the migration of 5TGM1 cells in vitro was also assessed using a 24‐hour transwell assay in which primary murine BM stromal cell‐conditioned medium was uses as the chemoattractant. As shown in Figure 3C, Samsn1 was found to have no effect on the migration of 5TGM1 cells toward this stimulus after 24 h (*p* = 0.8565, paired t test). Furthermore, the adhesion of 5TGM1 cells to BM endothelial cells (an important preliminary process for BM extravasation) was shown to be unaffected by Samsn1 expression (*p* = 0.1267, paired t test; Figure 3D). Taken together, these data suggest that while Samsn1 does not inhibit the homing of 5TGM1 cells to the BM, it does inhibit the outgrowth of disseminated MM PC within the BM microenvironment and prevents overt metastases from forming.

### 3.3 SAMSN1 affects neither the proliferation nor migration of human myeloma cells in vitro

**FIGURE 4:**
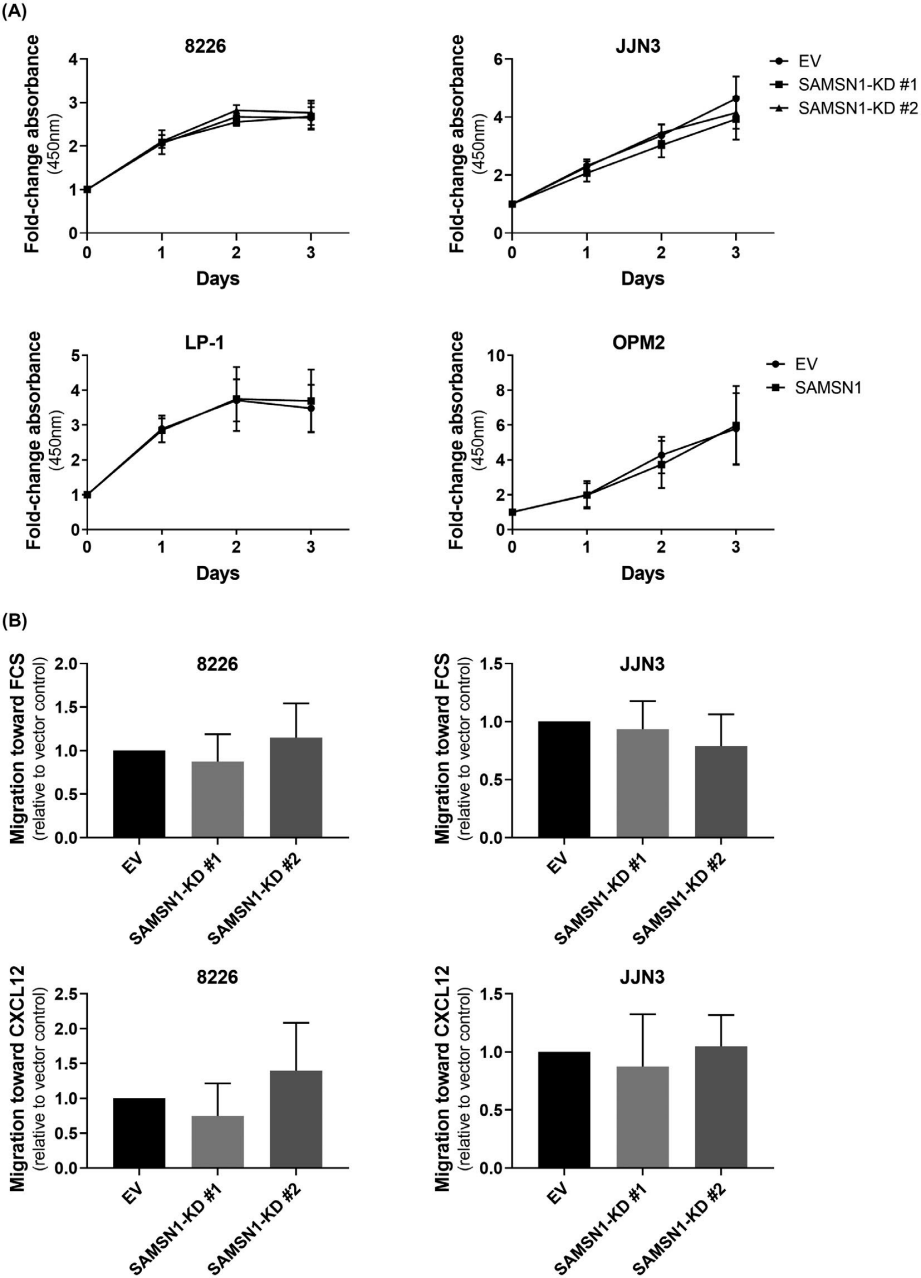
SAMSN1 expression does not affect the proliferation or migration of HMCLs in vitro. (A) The proliferation of SAMSN1‐knockdown (KD) versus EV control RPMI‐8226 and JJN3 cells, and SAMSN1‐transgene versus EV control LP‐1 and OPM2 cells, was measured over 3 days by a WST‐1 assay. Results were expressed as fold‐change in absorbance (450 nm) normalized to day 0. Graphs depict the mean ± SD of three independent experiments. *p* > 0.05, two‐way ANOVA with Sidak's multiple comparisons test. (B) Migration of SAMSN1‐knockdown (KD) versus EV control RPMI‐8226 and JJN3 cells toward either 20% FCS or 100 ng/ml CXCL12 was assessed in a transendothelial migration assay. Results are expressed relative to the EV control cells. Graphs depict the mean +SD of three or more independent experiments. *p* > 0.05, one‐way ANOVA with Tukey's multiple comparisons test

In order to discover whether the tumor suppressor properties of Samsn1 were also apparent in human myeloma cells, a combination of CRISPR‐Cas9 mutation and viral transgenesis was used to generate 4 different human myeloma cell lines (HMCLs) with matched SAMSN1‐high and SAMSN1‐low/null expression (Supplementary Figure S1). Neither knocking out SAMSN1 expression in the SAMSN1‐high cell lines RPMI‐8226 and JJN3, nor overexpressing SAMSN1 in the SAMSN1‐low cell lines OPM2 and LP1 cells had any significant effect on the short‐term proliferation rates of these cell lines in vitro (Figure 4A). Furthermore, reducing SAMSN1 expression in RPMI‐8226 and JJN3 cells did not affect the relative migration of either of these cell lines towards either FCS or CXCL12 in transwell assays (Figure 4B).

### 3.4 SAMSN1 does not affect the growth of human myeloma cell tumors in vivo

**FIGURE 5:**
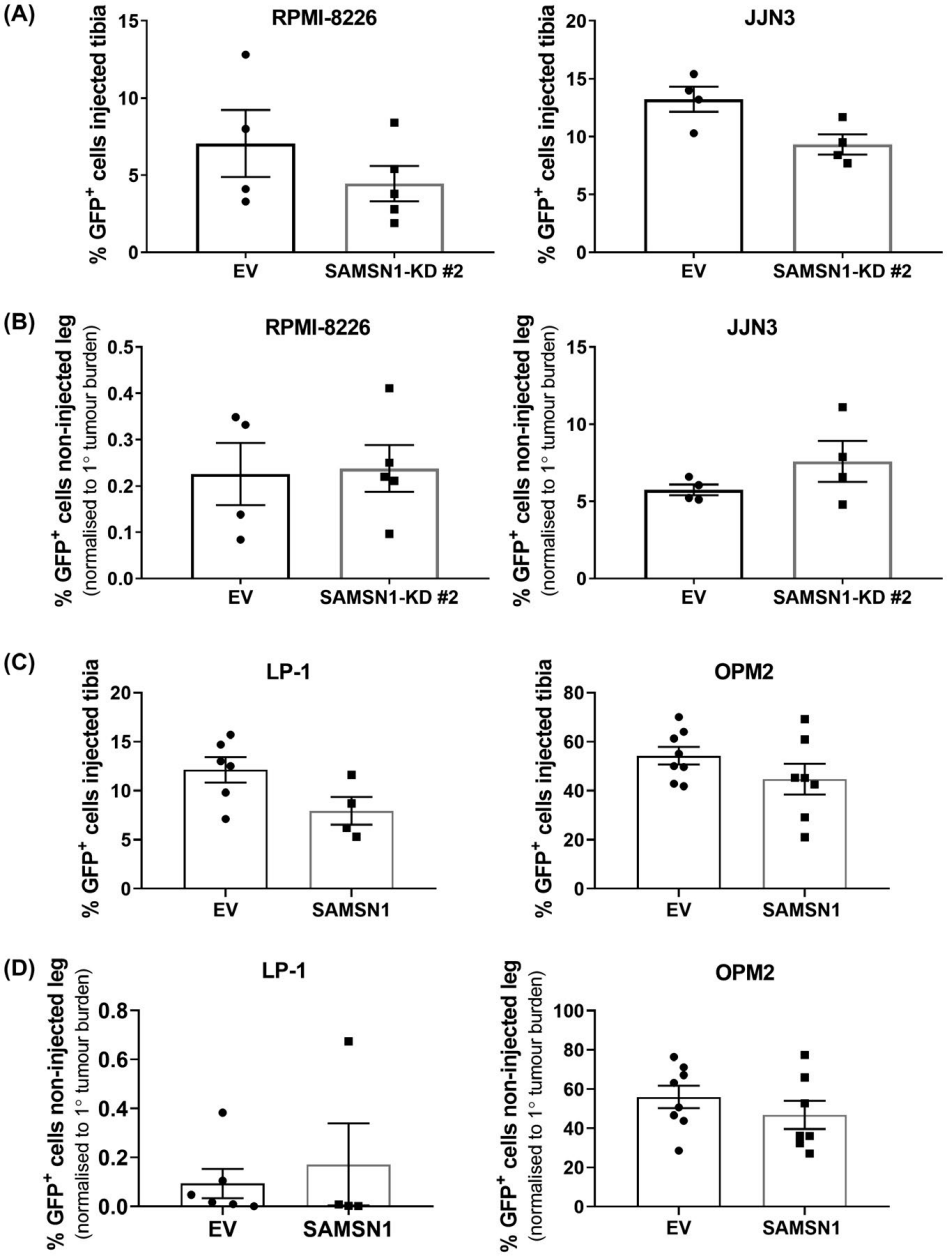
SAMSN1 expression does not affect the primary or metastatic tumor growth of HMCLs in vivo. (A and B) SAMSN1‐KD #2 or EV control RPM1‐8226 and JJN3 HMCLs were injected into the left tibiae of NSG mice. Tumors were allowed to develop in mice inoculated with RPM1‐8226 or JJN3 HMCLs over 5 or 3 weeks, respectively. (A) The percentage of GFP^+^ SAMSN1‐KD and EV RPM1‐8226 or JJN3 cells in the BM of the injected tibiae were determined by flow cytometry at the experimental endpoint. (B) The percentage of GFP^+^ SAMSN1‐KD and EV RPM1‐8226 or JJN3 cells in the BM of the non‐injected, contralateral femora and tibiae was determined by flow cytometry at the experimental endpoint. Results were normalized to primary tumor burden. (C and D) SAMSN1‐transgene expressing (SAMSN1) or empty vector (EV) control LP‐1 and OPM2 cells were injected into the left tibiae of NSG mice and disease was allowed to develop over 8 or 3 weeks, respectively. (C) The percentage of GFP^+^ SAMSN1 and EV LP‐1 or OPM2 cells in the BM of the injected tibiae was determined by flow cytometry at the experimental endpoint. (D) The percentage of GFP^+^ SAMSN1 and EV LP‐1 or OPM2 cells in the BM of the non‐injected, contralateral femora and tibiae were determined by flow cytometry at the experimental endpoint. Results were normalized to primary tumor burden. Graphs depict the mean ± SEM of *n* = 4‐5 mice per cell line from one experiment (A and B), mean ±SEM of *n* = 4‐8 mice per cell line from two independent experiments (C and D). *p* > 0.05, Mann–Whitney *U* test (A‐D)

Human myeloma cell lines can be studied in vivo but only successfully engraft in immune deficient mice such as the NOD.Cg‐Prkdc*^scid^*Il2rg*^tmlWjl^*/SzJ (“NSG”) mice. Xenografts were conducted to ascertain whether SAMSN1 expression affected the intramedullary growth of human MM cells in long bones, and/or whether SAMSN1 would reduce the ability of the myeloma cells to form distal metastases. In separate experiments, all four paired GFP^+^ human MM cell lines were injected directly into the tibiae of NSG mice and the establishment and progressive growth of both the primary tumors in the injected legs, and metastatic tumors in the contralateral legs, were measured by enumerating GFP^+^ tumor cells at experimental endpoints. Knocking out SAMSN1 expression in either of the two SAMSN1‐high cell lines had no effect on the ability of the tumors to grow in either leg. There was no difference between the primary tumor burden within the injected tibiae of mice inoculated with SAMSN1‐KD cells compared to mice inoculated with the EV control cells for either the RPMI‐8226 or JJN3 HMCLs (*p* > 0.05, Mann–Whitney *U* test; Figure 5A). In addition, reduced SAMSN1 expression did not affect the number of metastatic RPMI‐8226 or JJN3 tumor cells in the BM of the non‐injected legs of the mice (*p* > 0.05, Mann–Whitney *U* test; Figure 5B). Transgene expression of SAMSN1 in the two SAMSN1‐low/‐null cell lines also had no effect on the capacity of the injected cells to establish and proliferate within the injected tibiae or to establish metastatic tumors in the non‐injected legs. For LP‐1 cells, SAMSN1 overexpression did not significantly affect tumor burden either in the injected tibia (*p* = 0.0667, Mann–Whitney *U* test; Figure 5C), or the non‐injected leg (*p* = 0.5273, Mann–Whitney *U* test; Figure 5D). Of note, the LP‐1 cell line was only weakly metastatic, which is consistent with a previous report that LP‐1 cells do not migrate in vitro.^51^ In contrast, OPM2 cells were found to be highly metastatic, but neither the primary (*p* = 0.2319, Mann–Whitney *U* test; Figure 5C) nor metastatic (*p* = 0.3969, Mann–Whitney *U* test; Figure 5D) tumor burden was found to differ between the mice injected with the OPM2‐SAMSN1 cells and those injected with the OPM2‐EV control cells.

### 3.5 Samsn1 only reduces the growth of 5TGM1 tumors in Samsn1‐null mice

**FIGURE 6:**
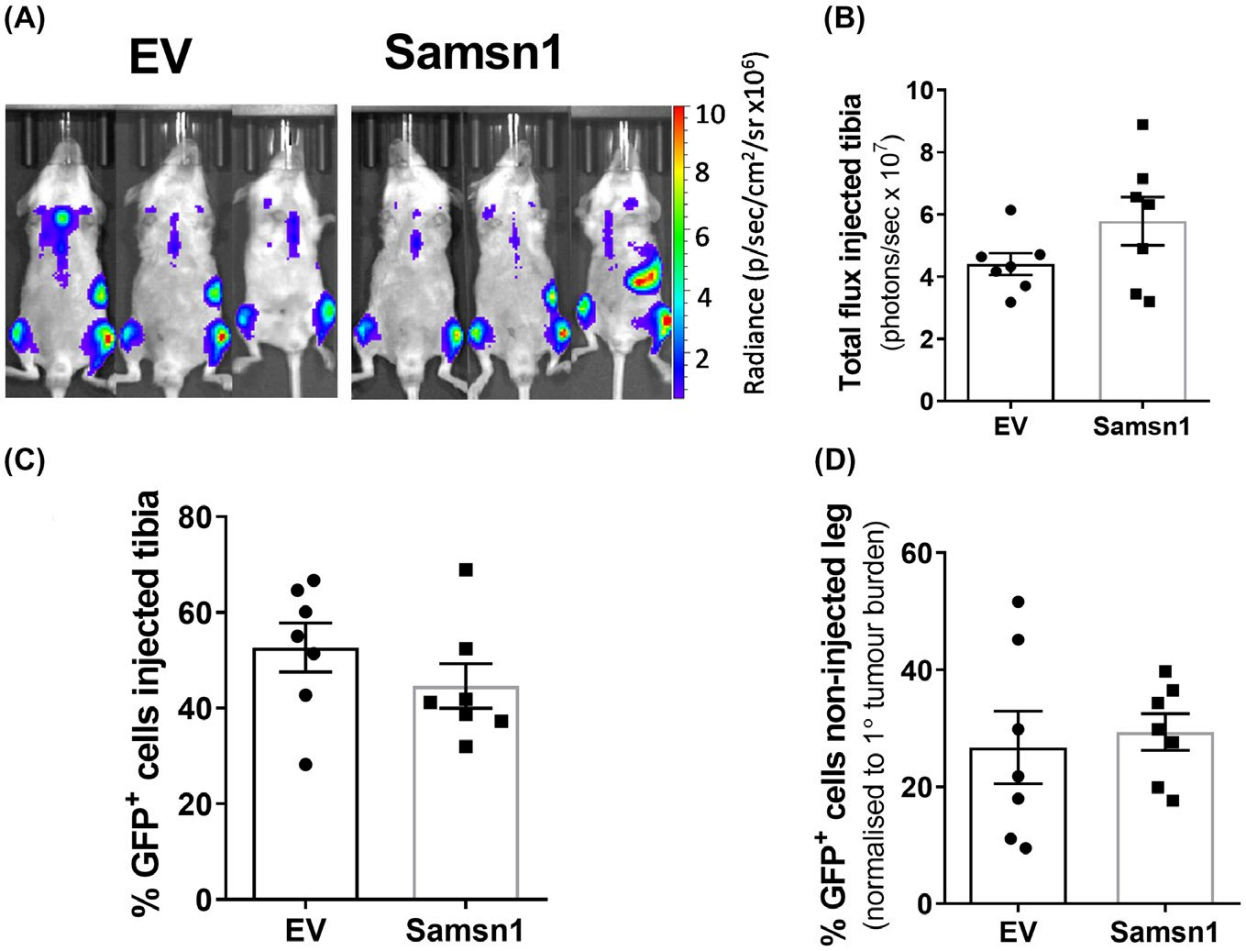
Samsn1 expression in 5TGM1 cells does not affect tumor growth following i.t. injection into NSG mice. Samsn1‐expressing or EV control 5TGM1 cells were injected into the left tibiae of NSG mice and disease was allowed to develop for 23 days. (A) Tumor burden was measured by BLI on day 23 post‐tumor cell inoculation and representative ventral scans of the mice are shown. (B) The total flux from the injected leg was quantitated from the ventral BLI scans. (C) The percentage of GFP^+^ Samsn1‐overexpressing/EV 5TGM1 cells in the BM of the injected tibiae was determined by flow cytometry at the experimental endpoint. (D) The percentage of GFP^+^ Samsn1‐overexpressing/EV 5TGM1 cells in the BM of the non‐injected hind legs was determined by flow cytometry at the experimental endpoint. Results were normalized to primary tumor burden. Graphs depict the mean ±SEM of n = 7 mice per cell line from two independent experiments. *p* > 0.05, Mann–Whitney *U* test

**FIGURE 7:**
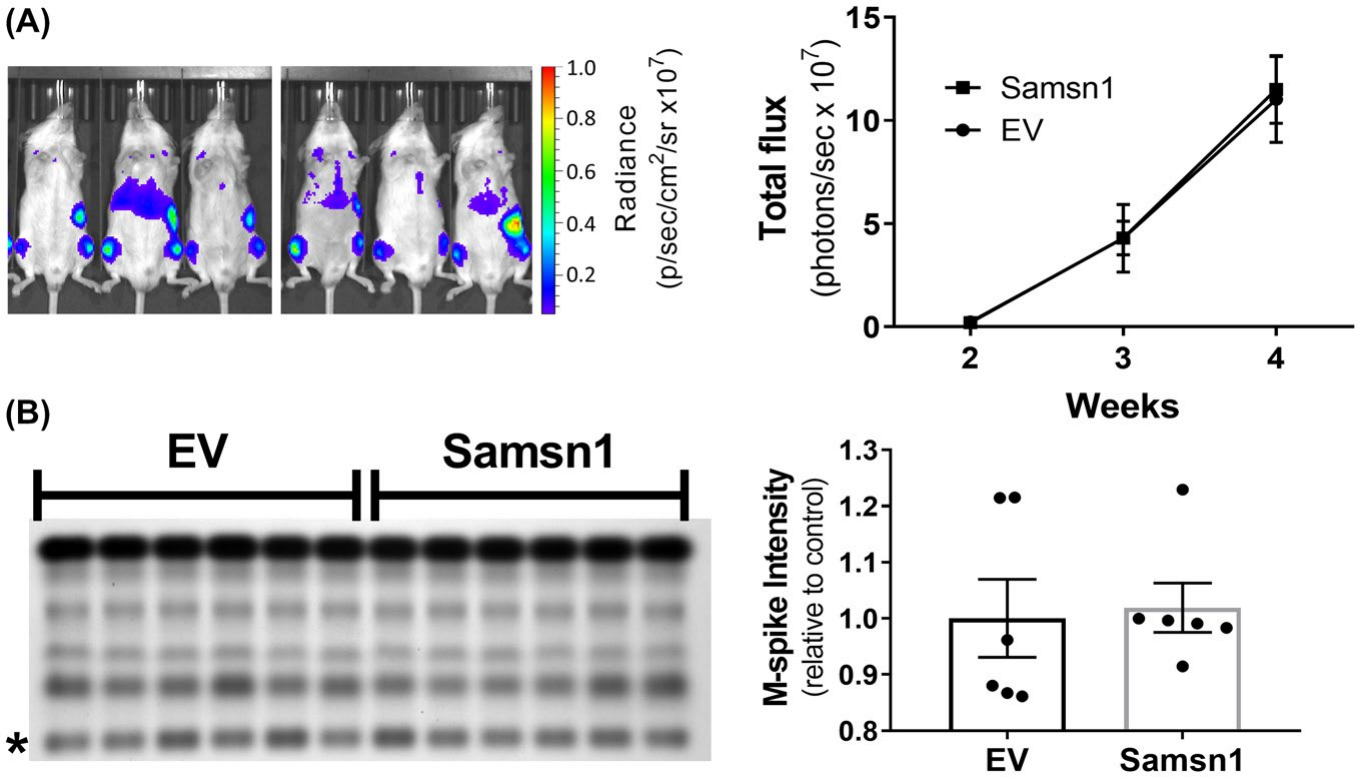
Samsn1 expression in 5TGM1 cells does not affect tumor growth following i.v. injection into NSG mice. Samsn1‐expressing or EV control 5TGM1 cells were injected i.v. into NSG mice and disease was allowed to develop for 4 weeks. A, BLI of the mice injected with 5TGM1‐Samsn1 or 5TGM1‐EV cells was performed weekly from week 2. Representative ventral scans after 4 weeks and the quantitated total flux from the ventral scans over time are shown. B, SPEP was performed on sera collected from the mice after 4 weeks. The SPEP gel and the M‐spike (*) intensity expressed relative to the EV control (right) are shown. Graphs depict the mean ± SEM of n = 6 mice per cell line from one experiment. *p* > 0.05, two‐way ANOVA with Sidak's multiple comparisons test (A) or Mann–Whitney *U* test (B)

SAMSN1 overexpression in HMCLs did not significantly inhibit metastasis following intratibial (i.t.) injection of tumor cells in vivo, which contrasted with the significant suppression of metastasis caused by Samsn1 re‐expression in the 5TGM1/KaLwRij i.t. model of MM. It was hypothesized that these conflicting findings may be attributable to the use of immunodeficient NSG mice in the HMCL xenograft models. To test this, NSG mice were inoculated with Samsn1‐expressing or EV control 5TGM1 cells by i.t. injection and primary and metastatic tumor burdens measured by BLI and flow cytometry after 23 days. Consistent with the results in KaLwRij mice, Samsn1 did not affect the growth of primary tumors in the injected tibia of NSG mice, as determined by BLI (*p* = 0.1649, Mann–Whitney *U* test; Figure 6A,B) and flow cytometry (*p* = 0.2319, Mann–Whitney *U* test; Figure 6C). However, the metastatic tumor burden in the non‐injected, contralateral hind leg was not reduced in NSG mice inoculated with 5TGM1‐Samsn1 cells compared to those inoculated with 5TGM1‐EV cells, as determined by flow cytometry (*p* = 0.4634, Mann–Whitney *U* test; Figure 6D). In addition, Samsn1 expression did not inhibit the growth of 5TGM1 cells following i.v. injection into NSG mice, as measured by whole animal BLI (*p* = 0.9108, Mann–Whitney *U* test; Figure 7A) and serum protein electrophoresis (SPEP) quantitation of the M‐spike (monoclonal antibody secreted by the tumor cells) (*p* = 0.3095, Mann–Whitney *U* test; Figure 7B). Hence, the previously observed ability of Samsn1 to inhibit the outgrowth of disseminated 5TGM1 cells in immunocompetent KaLwRij mice (Figure 2, and Noll, et al.^37^) was lost in immunodeficient NSG mice, suggesting that the tumor suppressor effect of Samsn1 in MM PCs is dependent on the presence of a functional immune system.

**FIGURE 8:**
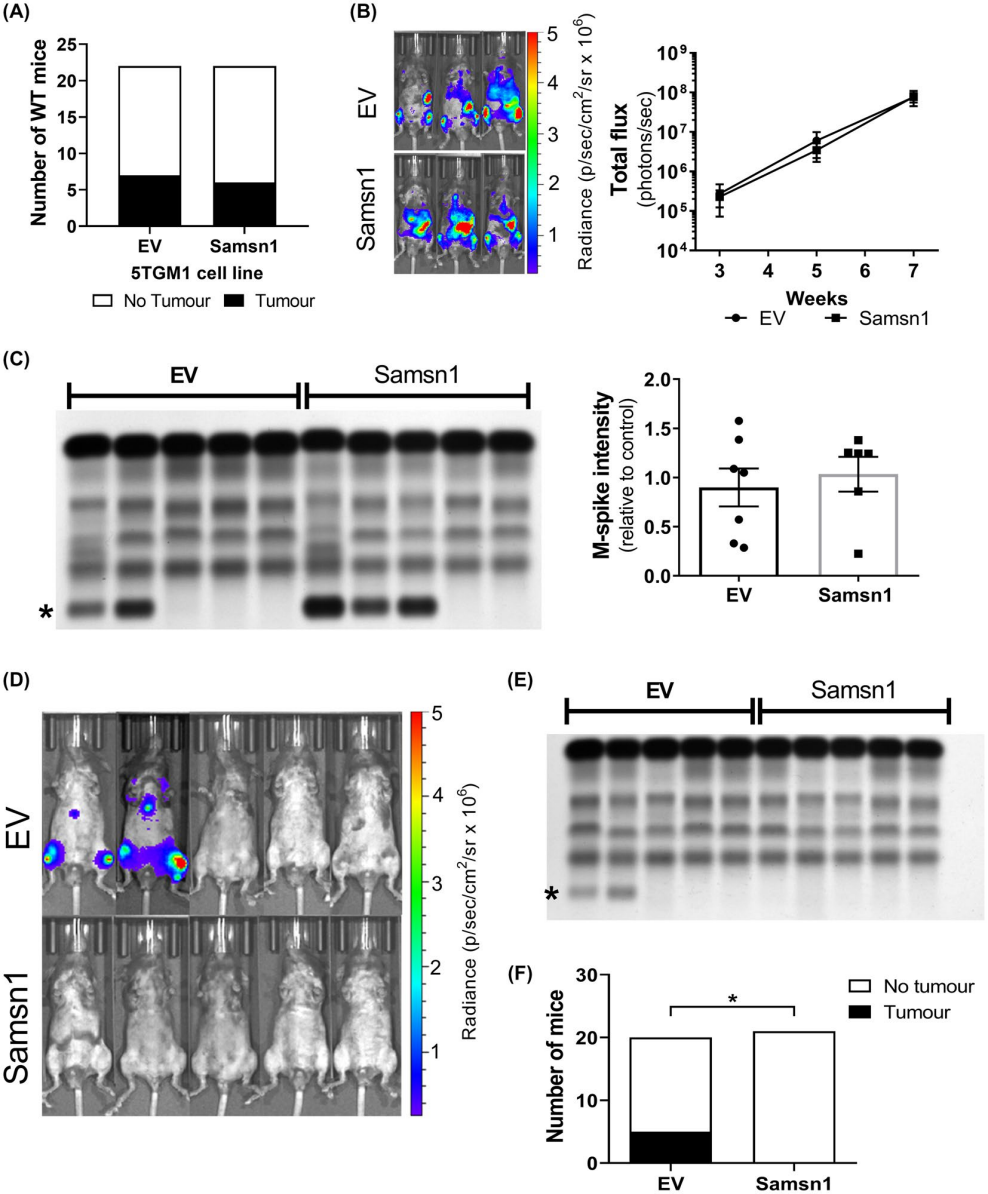
Samsn1 expression in 5TGM1 cells inhibits MM tumor development in immunocompetent C57BL/6/*Samsn1* ^−/−^ mice but not in C57BL/6 (Samsn1^+/+^) mice. (A‐C) Samsn1‐expressing or EV control 5TGM1 cells were injected i.v. into C57BL/6 (Samsn1^+/+^) mice and tumor was allowed to develop for 7 weeks (*n* = 22 mice per cell line). Tumor burden was measured by BLI at weeks 3, 5 and 7 post‐tumor cell inoculation and by SPEP at week 7. (A) The numbers of C57BL/6 mice inoculated with 5TGM1‐Samsn1 or 5TGM1‐EV cells that were tumor‐bearing by week 7, as determined by BLI and SPEP. (B) For tumor‐bearing mice, representative BLI ventral scans at 7 weeks and the quantitated total flux from the ventral scans over time are shown. (C) Representative SPEP gel of serum samples from tumor‐bearing and non‐tumor‐bearing mice (left, * = M‐spike) inoculated with 5TGM1‐EV or 5TGM1‐Samsn1 cells are shown. For tumor‐bearing mice, the quantitated M‐spike intensities are shown. Graphs depict the mean ± SEM of *n* = 6‐7 tumor‐bearing mice per cell line from two independent experiments (B and C). *p* > 0.05, Fisher's exact test (A), two‐way ANOVA with Sidak's multiple comparisons test (B) or Mann–Whitney *U* test (C). (D‐F) Samsn1‐expressing or EV 5TGM1 cells were injected i.v. into C57BL/6/*Samsn1* ^−/−^ mice and tumor was allowed to develop for 7 weeks. Tumor burden was measured by BLI at weeks 3, 5 and 7 post‐tumor cell inoculation and by SPEP at week 7. (D) Representative ventral BLI scans of mice inoculated with 5TGM1‐EV or 5TGM1‐Samsn1 cells at week 7 are shown. (E) A representative SPEP gel containing serum samples from the mice included in (D) is shown (* = M‐spike). (F) The proportion of tumor‐bearing mice at 7 weeks post inoculation with either 5TGM1‐Samsn1 or 5TGM1‐EV cells, as determined by BLI and SPEP. Graph depicts *n* = 20‐21 mice per cell line from two independent experiments. *p < 0.05, Fisher's exact test

To test whether the presence of a functional adaptive immune system *per se* in a murine recipient was sufficient to restore the tumor suppressor effect of Samsn1 previously observed in immunocompetent KaLwRij mice,^37^ Samsn1 expressing 5TGM1 cells were injected into the non‐syngeneic (genetically nonidentical) but immunocompetent C57BL/6 mice. Previous results in our laboratory have shown that 5TGM1 cells are capable of forming bone marrow tumors in C57BL/6 mice, albeit at a significantly reduced penetrance (circa 25% compared to 95% for KaLwRij mice) and longer latency (7 weeks until maximum tolerated tumor burden compared to 4 weeks for KaLwRij mice) (data not shown). Samsn1‐expressing or EV control 5TGM1 cells were injected i.v. into C57BL/6 mice, which were then monitored for tumor development over 7 weeks. Samsn1 expression in the 5TGM1 tumor cells was found not to affect tumor penetrance in mice, as determined by BLI or SPEP (*p* > 0.9999, Fisher's exact test; Figure 8A). In addition, of those C57BL/6 mice that developed tumor, tumor burden did not differ between the mice injected with 5TGM1‐Samsn1 cells and those injected with 5TGM1‐EV control cells, as measured by BLI (*p* = 0.9722, two‐way ANOVA with Sidak's multiple comparisons test; Figure 8B) and SPEP (*p* = 0.8357, Mann–Whitney *U* test; Figure 8C). These data suggest that Samsn1 does not suppress MM tumor development in the presence of a competent immune system in C57BL/6 mice. Hence, there may be unique features of the competent immune system in KalwRij mice that facilitate the suppression of 5TGM1‐Samsn1 tumor growth in this mouse strain.

Given that one of the most striking genetic differences between KaLwRij and C57BL/6 mice is that KaLwRij mice have a constitutive deletion of the *Samsn1* gene,^37^, ^38^ it was hypothesized that this abnormality may contribute to the unique ability of the KaLwRij mice to suppress 5TGM1‐Samsn1 cell engraftment. To test this, Samsn1‐expressing or EV 5TGM1 cells were injected intravenously into immunocompetent C57BL/6/*Samsn1* ^−/−^ mice (generated by backcrossing the KaLwRij‐derived *Samsn1* genomic deletion onto a C57BL/6 background for 10 generations). At 7 weeks post‐tumor cell inoculation, 5 of the 20 (25%) C57BL/*Samsn1* ^−/−^ mice injected with EV control 5TGM1 cells had developed tumor, whereas none of the 21 (0%) C57BL/6/*Samsn1* ^−/−^ mice that were injected with 5TGM1‐Samsn1 cells had any evidence of disease development, as determined by BLI and SPEP (Figure 8D‐F). This constituted a significant inhibition of tumor penetrance for 5TGM1‐Samsn1 cells compared to 5TGM1‐EV control cells in the C57BL/6/*Samsn1* ^−/−^ mice (*p* = 0.0207, Fischer's exact test). These data suggest that the previously demonstrated tumor suppressor effect of MM cancer cell‐intrinsic Samsn1 expression in vivo^37^ is dependent on the recipient mouse being both immunocompetent and *Samsn1* ^−/−^.

## 4 DISCUSSION

Our group and others have previously identified the adaptor protein SAMSN1 as a novel tumor suppressor in MM, the downregulation of which may promote MM development. This assertion was based on the finding that C57BL/KaLwRij mice, which unlike C57BL/6 mice can spontaneously develop MM, harbor a constitutive homozygous deletion of the *Samsn1* gene, suggesting that the loss of *Samsn1* may promote MM development in this strain.^37^, ^38^ In support of this, the introduction of Samsn1 into the KaLwRij‐derived MM PC 5TGM1 line was shown to abrogate tumor development in vivo.^37^ In relation to human MM, *SAMSN1* mRNA expression was found to be significantly reduced in the PCs of MM patients compared to healthy individuals, which was also consistent with SAMSN1 having a tumor suppressor role in MM patients.^37^, ^38^ The fact that *Samsn1* ^−/−^ KaLwRij mice only develop MM with late onset and incomplete penetrance (~1 in 200 mice over two years old)^39^, ^40^ suggests that the loss of *Samsn1* co‐operates with other genetic lesions to promote disease progression in these mice, and also potentially in patients.

While the abrogation of tumor development by Samsn1 in the 5TGM1/KaLwRij model suggested it was a potent tumor suppressor in MM, the mechanism(s) by which Samsn1 achieved this anti‐tumor effect was unclear. Although Samsn1 was shown to have an anti‐proliferative effect in normal B cells following BCR stimulation,^29^, ^31^ Samsn1 expression in 5TGM1 cells was previously found to cause only a modest reduction in proliferation and then only when the tumor cells were co‐cultured with bone marrow stromal cells (BMSCs) in vitro.^37^ This suggested that there may be a mechanism, other than intrinsic inhibition of MM PC proliferation, by which Samsn1 inhibits tumor growth in vivo.

Here we report that Samsn1 did not affect the in vitro migration or the in vivo BM homing of 5TGM1 cells. Notably, following the orthotopic intratibial delivery of 5TGM1 cells, Samsn1 was found to inhibit the growth of metastatic, but not primary, tumors in the BM of KaLwRij mice. The observation that Samsn1 only limited the outgrowth of 5TGM1 cells when relatively few had seeded the BM, suggested that Samsn1 may promote BM microenvironment‐mediated control of MM PC outgrowth.^52^ Neither the upregulation of SAMSN1 by transgenic overexpression, nor the downregulation of SAMSN1 by CRISPR‐mediated genome editing, affected the growth of metastatic tumors of human MM cell lines within the BM of immunodeficient NSG mice. This contrasted with the significant inhibition of disseminated 5TGM1 cell outgrowth in the BM of immunocompetent KaLwRij mice. Crucially, it was also revealed that the ability of Samsn1 to suppress the outgrowth of disseminated 5TGM1 cells in the BM was absent in immunodeficient NSG mice, suggesting that functional immune cells are required for the tumor suppressor effect of Samsn1 in vivo. Samsn1 was subsequently found to inhibit 5TGM1 cell growth in immunocompetent C57BL/6/*Samsn1* ^−/−^ mice but not in immunocompetent C57BL/6/Samsn1^+/+^ mice. These findings are consistent with Samsn1 only promoting a graft rejection of 5TGM1 cells from *Samsn1* ^−/−^ hosts in which Samsn1‐specific adaptive immune cells have not been eliminated by immune tolerance.

The evasion of immune destruction of cancer cells has long been recognised as a “emerging hallmark” of cancer.^53^ Patients with primary immunodeficiencies, or on long‐term immunosuppressive therapies were known to have higher incidences of certain cancers.^54^, ^55^ Landmark observations in murine models of cancer provided further evidence of the existence of a protective immune surveillance mechanism. Mice deficient in RAG2, which lack B and T effector immune cells, had more spontaneous carcinogen induced tumors than wild type mice,^56^ a phenomenon that was also seen in mice lacking the receptor for the key immune cytokine interferon‐γ,^57^ and in mice deficient for perforin, a key T‐cell and NK cell effector protein.^58^ Immune surveillance is proposed to be responsible for the elimination of the majority of emergent cancerous cells prior to them becoming overt tumors. The host immune response to cancer is now known to not only protect against cancer initiation, but also sculpt the character of the tumors that do emerge and is comprised of three distinct phases: elimination, equilibrium and escape. Early stages of the elimination phase involve NK, NKT and γδ Tcells mediated killing of cancer cells. Priming of T cells, the generation of T cells reactive to specific tumor antigens, and the homing of CTLs (cytotoxic T lymphocytes) to the tumor site are part of the late stage of the elimination phase. During the elimination phase non‐self antigens (often termed cancer‐cell specific neoantigens) are processed and presented on MHC class I molecules on the surface of the cancer cells.^59^ A long period of equilibrium or immune‐mediated tumor dormancy can then ensue, wherein the immune system sculpts or “immunoedits” the cancer.^60^ This equilibrium phase is associated with cancer cells beginning to lose their immunogenicity and the emergence of immune resistance. Ultimate escape from immune surveillance involves multiple mechanisms such as the loss of tumor‐specific antigen expression, the downregulation of MHC Class I or other costimulatory molecules, and the generation of an immune suppressive tumor microenvironment (TME). Importantly, therapeutics aimed at alleviating immune suppression, such as anti‐CTLA‐4 and anti‐PD1 antibodies,^61^ have shown persistent clinical responses in a number of cancer types.

The rejection of Samsn1‐expressing 5TGM1 myeloma cancer cells is characteristic of the classic elimination and equilibrium phases of control of cancer by immune surveillance. The assumption is that Samsn1 neoantigens are being processed and presented on the cancer cell surface bound to MHC class I molecules for recognition by previously primed Samsn1‐epitope specific effector T cells. Our own attempts to definitively show an immune response against Samsn1 neoantigens have proved inconclusive. We saw no evidence of increases in T, NK, NKT cell activation by flow cytometric enumeration of CD86^+^ cells in peripheral blood, spleen or bone marrow following 5TGM‐Samsn1 cell inoculation of KaLwRij mice (data not shown). Attempts to evaluate the cytolytic T lymphocyte activity of splenic CD8^+^ effector T cells isolated from KaLwRij mice repeatedly inoculated with 5TGM1‐Samsn1 cells were hampered by very low and inconsistent levels of cell lysis (data not shown). To ascertain whether there was a B cell mediated humoral response to Samsn1 neoantigens, the production of anti‐Samsn1 antibodies following exposure to 5TGM1‐Samsn1 cells was assessed in vivo. KaLwRij, C57BL/6/*Samsn1* ^−/−^ and C57BL/6/*Samsn1* ^+/+^ mice were twice inoculated with either 5TGM1‐Samsn1 or control 5TGM1‐EV cells and the presence of anti‐Samsn1 antibodies in their serum was then determined by Western blot (data not shown). No anti‐Samsn1 antibodies were detected in the serum of control C57BL/6/*Samsn1* ^+/+^ mice (*n* = 3). Neither were anti‐Samsn1 antibodies detected in any of three KaLwRij mice. An inconsistent result was observed in C57BL/6/*Samsn1* ^−/−^ mice where only 1 of 5 animals showed low levels of detectable anti‐Samsn1 antibodies (data not shown).

The observed lack of growth of 5TGM1‐Samsn1 in KaLwRij mice was limited to the 4 week time scale of this cancer model. It would be interesting to extend the experiment and see if 5TGM1 tumors eventually become established. It may be anticipated that the disease will eventually relapse after an unknown period of immune equilibrium. Secondary transplants with any emergent 5TGM1‐Samsn1 cancers could be performed, and they may well have undergone immunoediting and could be less immunogenic and may no longer rejected by *Samsn1* ^−/−^ hosts. Such a result would be similar to the loss of immunodominant rejection antigens that has been observed when other chemical‐ or oncogene‐induced cancers established in immunodeficient mice were transplanted into immune competent recipients.^62^, ^63^ Another commonly used approach for confirming specific immune cell involvement in the rejection of 5TGM1‐Samsn1 cancer cells would be the co‐administration of neutralizing antibodies such as either anti‐CD4 and/or anti‐CD8 to target T‐cell subsets, or anti‐asialoGM1/anti‐NK1.1 to target NK cells,^64^, ^65^ some of these antibodies should enable the 5TGM1‐Samsn1 cancers to become established in *Samsn1* ^−/−^ hosts.

Immunocompetent murine syngeneic transplantation tumor models are indispensable for the study of the complex interactions between cancer and immune cells and for testing novel immunotherapies. The use of immunocompetent cancer models can be complicated by immune‐mediated graft rejection directed toward non‐disease related neo‐antigens expressed by the cancer cells. For instance, to enable the growth of the tumor to be tracked in vivo and *ex vivo*, it is common for the syngeneic tumor cells to be engineered to overexpress reporter proteins, such as GFP and luciferase. Studies have shown that the expression of some of these xenogeneic proteins can generate reporter‐specific CTL responses in some immunocompetent tumor models, which limits tumorigenesis, and metastasis.^66^, ^67^, ^68^, ^69^, ^70^, ^71^, ^72^ The same phenomenon has also become even more apparent when using syngeneic tumor cell lines that express the highly antigenic bacterial Cas9 protein (a commonly used genetic modification enzyme).^71^, ^72^ The immunogenicity of the foreign protein is influenced by several factors, including the expression level of the protein, the cell type expressing the protein, and the genetic background of the host.^73^, ^74^ This is evidenced by the enhanced immune response to GFP displayed by Balb/c mice compared to C57BL/6 mice.^69^, ^75^, ^76^ However, the expression of reporter proteins in syngeneic cancer cells does not prevent tumor growth in many immunocompetent models,^77^, ^78^, ^79^, ^80^ including the 5TGM1/KaLwRij model in which the overexpression of GFP and luciferase does not prevent aggressive tumor development.^37^, ^81^, ^82^

It was unexpected that expression of Samsn1, which is a foreign protein in *Samsn1* ^−/−^ KaLwRij mice, would elicit an immune response that was capable of completely abrogating tumor growth in vivo. Our findings parallel recent observations of Ozturk, et al,^83^ where adoptive transfer of Eµ‐TCL1 chronic lymphocytic leukemia (CLL) tumor cells into syngeneic mice harboring a variety of single gene knockouts was conducted. CD8+ T‐cell mediated graft rejection was frequently observed when the specific gene that was knocked out in the recipient mouse happened to be expressed by the injected tumor cells. This was exemplified by the observation that Granulin (Grn) positive tumor cells were completely rejected from *Grn* ^−/−^ recipient mice but engrafted readily in wild type mice. By contrast *Grn* ^−/−^ splenocytes could engraft into *Grn* ^−/−^ hosts. Furthermore, rejection of *Grn* ^+/+^ splenocytes from *Grn* ^−/−^ mice was accelerated following previous exposure of *Grn* ^−/−^ mice to *Grn* ^+/+^ tumor cells, consistent with immunological memory. CD8^+^ T‐cell reconstitution experiments in B‐ and T‐cell deficient RAG2^−/−^ mice further confirmed the CD8^+^ T‐cell‐mediated nature of the rejection of *Grn* ^+/+^ tumors cells. Together with our results, this suggests that mismatches in gene expression between recipient mice and administered tumor cells need to be carefully considered when designing and interpreting in vivo tumor engraftment studies. Indeed, even a cursory review of published literature reveals many examples of preclinical cancer models where primary tumor growth and/or the degree of metastasis are significantly reduced following administration of syngeneic cancer cell lines into immunocompetent hosts that are genetic knockouts for a range of genes/proteins.^84^, ^85^, ^86^, ^87^, ^88^, ^89^, ^90^, ^91^, ^92^, ^93^ These include many studies with known mismatches in gene expression between the cancer cells and the recipient knockout mice,^85^, ^87^, ^88^, ^89^, ^90^, ^92^ where immune rejection of the allografts could be contributing to the significant reductions in tumor growth observed. Some of the results of Shiuan, et al,^90^ investigating the effects of host EphrinA1‐deficiency on breast cancer progression bear a striking similarity to our own observations concerning Samsn1 and myeloma.  4T1 breast cancer cells were injected orthotopically into the mammary glands of either wild type or *EphrinA1* knockout Balb/c mice. Despite no difference in the growth rates of the primary tumors between wildtype and knockout recipients, there was a significant reduction in the number of lung metastases in the *EphrinA1*‐KO mice. Reduced lung colonization of 4T1 cells was seen in *EphrinA1*‐KO mice following tail vein administration of the cancer cells, and resected primary tumors also grew back more slowly in *EphrinA1*‐KO mice. The authors had previously noted that 4T1 cells display abundant expression of EphrinA1,^94^ yet whether EphrinA1‐expressing 4T1 cells would be recognized as non‐self by the adaptive immune system of *EphrinA1*‐KO mice was not considered. Other notable recent examples include: the significantly slower growth of the Nox1 expressing B16‐F10 melanoma and MC38 colorectal cells in *Nox1*‐knockout mice,^92^ and the 87% reduction in mean size of OPN‐expressing MC38 tumors in *OPN*‐knockout mice.^89^

MM cancer cells are dependent on bone marrow microenvironmental factors for their survival, growth, and dissemination, and knockout mouse models are often used as tools to investigate the contribution of host derived factors on myeloma disease course.^95^, ^96^, ^97^, ^98^ Some of these approaches utilize single gene knockouts on a RAG2^−/−^ background to avoid the complication of any B‐ nor T‐cell mediated tumor cell rejection^96^, ^97^ but others retain a fully functional adaptive immune system. Given the caveats outlined in this study, it is important for genetic knockout experiments to be complemented by molecular interference/inhibition of protein function wherever possible.

SAMSN1 remains a candidate tumor suppressor protein in MMPCs, and its absence has modest yet significant effects on the cell proliferation of both non‐malignant B cells^29^, ^30^, ^31^ and the 5TGM1 murine myeloma PC line.^37^, ^38^ However, a comprehensive assessment of the biological effects of modulating SAMSN1 expression in 5 MM cell lines across 4 strains of mice have led to a reassessment of the potency of its tumor suppressor activity, and we now believe that loss of its expression is only likely to play a major role in MM pathogenesis in combination with other dysregulated/mutated genes.

## CONFLICT OF INTEREST

The authors declare no conflicts of interest.

## AUTHOR CONTRIBUTIONS

N. Friend, V. Panagopoulos and D. Hewett conducted and planned the experimental work; V. Panagopoulos, J. Noll, K.Vandyke, K. Mrozik, and S. Fitter optimized experimental models and gave critical feedback on experiments and manuscript; A. Zannettino supervised and guided the experimental work; N. Friend, D. Hewett and A. Zannettino wrote the manuscript.

## Supporting information

Fig S1

Supplementary Material
