# Supplementary figures and images for: Characterization of the role of Samsn1 loss in multiple myeloma development
Source: FASEB Bioadv. 2020 Aug 5;2(9):554–72. doi: 10.1096/fba.2020-00027 (PMC7475304; doi:10.1096/fba.2020-00027)

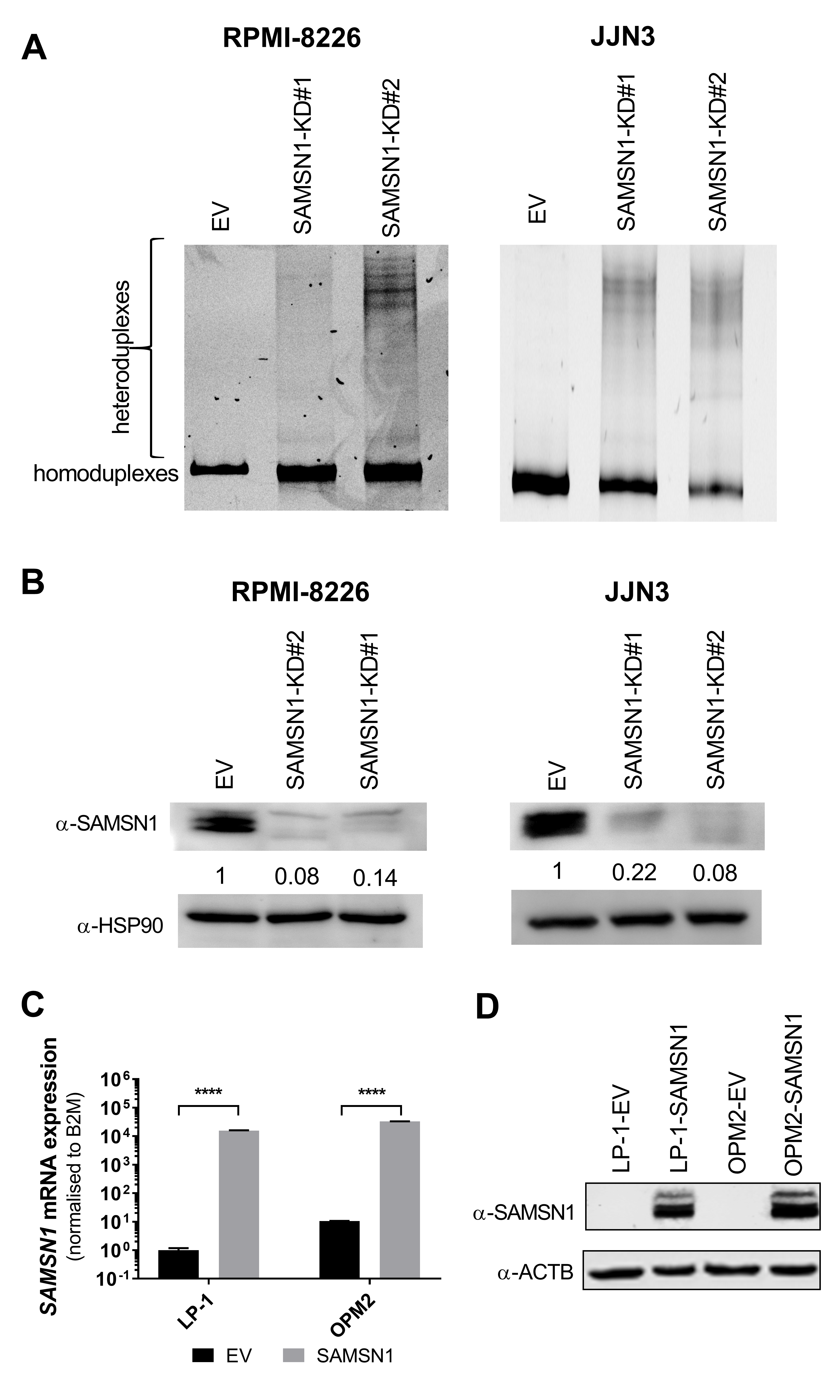

Supplement: Supplementary file 1 — Fig S1 [file FBA2-2-554-s001.tif]
