# Supplementary material for: Characterization of the role of Samsn1 loss in multiple myeloma development
Source: FASEB Bioadv. 2020 Aug 5;2(9):554–72. doi: 10.1096/fba.2020-00027 (PMC7475304; doi:10.1096/fba.2020-00027)
Supplement: Supplementary file 2 — Supplementary Material [file FBA2-2-554-s002.pdf]

Supplementary Figure Legend

Supplementary Figure 1. Generation of paired HMCLs with or without SAMSN1 expression. (A-B) CRISPR-Cas9 targeting of *SAMSN1* using doxycycline (dox)-inducible guide RNA (gRNA) lentiviral vectors in RPMI-8226 and JJN3 human myeloma cell lines. These cells were transduced with a lentiviral, dox-inducible gRNA expression vector encoding either one of two gRNAs (#1 and #2) targeting exon 4 of *SAMSN1* or no gRNA (an empty vector (EV) control) (**A**) DNA was extracted from the SAMSN1-KD #1, SAMSN1-KD #2 and EV RPMI-8226 and JJN3 cells and the *SAMSN1* exon 4 region was amplified by PCR. The products were then analysed for the presence of indels using a heteroduplex mobility assay. Images of GelRed®-stained polyacrylamide gels (6%) showing separation of homoduplex and heteroduplex PCR products for the RPMI-8226 (left) and JJN3 (right) HMCLs are shown. (**B**) Representative Western blots for SAMSN1 in whole cell lysates from CRISPR-Cas9-targeted or control RPMI-8226 and JJN3 HMCLs from one of two independent experiments are shown. HSP90 was used as the loading control. The quantified densities of the SAMSN1 protein bands, which were normalised to HSP90 and expressed relative to the EV control cell line, are also shown. (**C-D**) SAMSN1-negative human myeloma cell lines LP-1 and OPM2 were transduced with either SAMSN1 encoding or empty vector retroviruses. (**C**) The expression of *SAMSN1* mRNA was assessed in LP-1 and OPM2 HMCLs transduced with a SAMSN1 expression vector or a control empty vector (EV) by RT-qPCR. Expression values were normalised to *B2M* mRNA levels and expressed relative to the LP-1-EV cells. (**D**) The levels of SAMSN1 protein were assessed in LP-1 and OPM2 HMCLs transduced with a SAMSN1 expression vector or a control EV by Western blot. ACTB was used as the loading control.
